# Observations of Nematicity, Dopants, and Zero-Bias Conductance Peaks for the Ca_0.9_La_0.1_FeAs_2_ Superconductor

**DOI:** 10.3390/nano13040622

**Published:** 2023-02-04

**Authors:** Jae-Joon Kim, Min Seok Park, Kyoung Seok Lee, Sang Hyun Joo, Jung Hoon Yoo, Dilip Bhoi, Byeong Hun Min, Kee Hoon Kim, Jinho Lee

**Affiliations:** 1 Department of Physics and Astronomy, Seoul National University, Seoul 08826, Republic of Korea; 2 Samsung Electronics Semiconductor R&D Center, Hwaseong-si 18448, Gyeonggi-do, Republic of Korea; 3 Center for Novel States of Complex Materials Research, Department of Physics and Astronomy, Seoul National University, Seoul 08826, Republic of Korea

**Keywords:** Fe-based superconductor, nematicity, zero-bias conductance peak, STM, topological superconductor

## Abstract

Ca_1−x_La_x_FeAs_2_ (CLFA112) belongs to a new family of Fe-based superconductors (FeSCs) and has a unique crystal structure featuring an arsenic zigzag chain layer, which has been proposed to be a possible two-dimensional topological insulator. This suggests that CLFA112 is a potential topological superconductor—a platform to realize Majorana fermions. Up to now, even a clear superconducting (SC) gap in CLFA112 has never been observed, and the SC properties of CLFA112 remain largely elusive. In this letter, we report the results of an atomic-scale investigation of the electronic structure of CLFA112 crystals using low-temperature scanning tunneling microscopy (STM). We revealed four different types of surfaces exhibiting distinct electronic properties, with all surfaces displaying dominating 2 × 1 surface reconstructions. On a Ca/La layer on top of an FeAs layer, a clear SC gap of ~12 mV was observed only at the crevices (vacancies) where the FeAs layer can be directly accessed. Remarkably, the FeAs termination layer displayed a dispersing nematic modulation both in real and q space. We also present peculiar zero-bias conductance peaks for the very As chain layer that is believed to exhibit a topological edge state as well as the influence of La dopants on the As chain layer.

## 1. Introduction

The discovery of FeSCs opened a new era in the research of high-transition-temperature superconductors [1] (HTSCs). Similar to cuprates, FeSCs have a layered structure consisting of SC iron pnictogen–chalcogen layers and spacer layers. Among many families of FeSCs discovered, the most common ones are 1111-family ReOFeAs (Re = rare earth), 122-family AeFe_2_As_2_ (Ae = alkaline earth), 111-family AFeAs (A = alkali metal), and 11-family Fe(Te,Se). The number of families is still growing, and, recently, new types of FeSCs with more complicated spacer layers have been synthesized, such as EuFeAs_2_ [2], (Ca,Re)FeAs_2_ (Re = La, Pr) [3,4], Ba_2_Ti_2_Fe_2_As_4_O [5], and Ca_10_(Pt_4_As_8_)(Fe_2−x_Pt_x_As_2_)_5_ [6,7]. A large number of available spacer layers provides a means of tuning SC properties through selection of the spacer layers. One critical question that remains to be answered is whether Fe-based superconductors share a common physics with Cu-based superconductors which exhibit unusual properties, including pseudogap phases [8,9,10], electronic modulations (often roughly referred to as charge density waves (CDWs) [11,12,13]), and broken Fermi surfaces [14,15,16,17].

**Figure 1:**
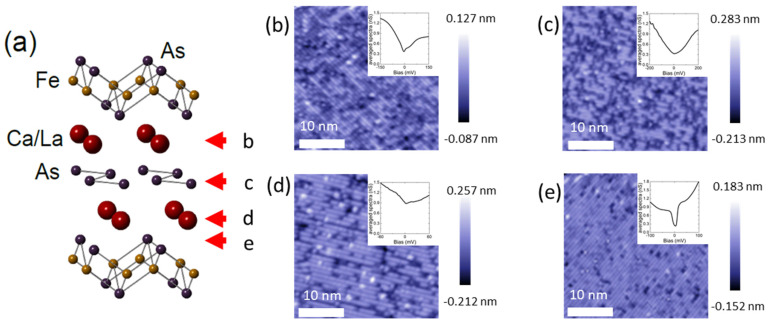
(**a**) Crystal structure of Ca_0.9_La_0.1_FeAs_2_. A 32 nm × 32 nm topographic image of (**b**) the Ca/La layer above the As chain layer, (**c**) the As chain layer, (**d**) the Ca/La layer above the FeAs layer, and (**e**) the FeAs layer. (Insets) Averaged spectra of each layer.

Recently, the 112 family, especially Ca_0.9_La_0.1_FeAs_2_ (CLFA112), with a T_c_ of around 45 K, has attracted substantial attention. Its crystal unit cell contains an additional layer of arsenic zigzag chains contrary to the conventional 122 family (Figure 1a), which might have significant effects on its electronic properties by introducing non-centrosymmetry. In addition to a similar Fermi surface to that of the 1111 family [18], it has been reported that another hole pocket exists at the center of the Brillouin zone and that there are small electron pockets at the X point consisting of As p orbitals [19,20,21]. More importantly, due to the strong spin–orbit coupling in the As zigzag chain layer, it has been proposed that the As chain layers could be two-dimensional topological insulators, hence making the material a potential topological superconductor which could host Majorana fermions [22]. This point was supported by the observation of linear dispersions forming a Dirac point close to the X point at about 90 mV below the Fermi energy [23].

## 2. Materials and Methods

We investigated near-optimally doped Ca_0.9_La_0.1_FeAs_2_ single crystals using low-temperature (LT) STM. The crystals were grown by the self-flux method, and their T_c_ was 46.5 K. A home-built high-vacuum, cryogenic-temperature STM with an ultra-low vibration system was used for all the measurements [24]. The crystal was cleaved in a cryogenic high vacuum and inserted into the STM head immediately and cooled to a base temperature of 4.2 K. In some cases, we rotated the cleaved sample in the cryogenic high vacuum to investigate different parts of the sample. Topographic images were taken in constant current mode, and the scanning tunneling spectroscopy was carried out using a standard lock-in method. (More details about the STM measurements are provided in the Supplementary Materials).

## 3. Results and Discussion

Figure 1b–e show high-resolution topographic images of the CLFA112 crystal, in which a dominating, stripe-like surface reconstruction can be seen. The insets show averaged spectra of each surface. At a glance, this reconstruction resembles the topographic images widely reported for 122 FeSCs or nickel-based superconductors with similar crystal structures, such as BaFe_2_As_2_ [25,26], SrFe_2_As_2_ [27,28,29,30,31], CaFe_2_As_2_ [32], Pr_1−x_Ca_x_Fe_2_As_2_ [33,34], and TlNi_2_Se_2_ [35]. In 122 FeSCs, the reported topmost surfaces are those of As in the FeAs layer or the 1/2 AE layer. Both topmost surfaces were reported to exhibit a 2 × 1 surface reconstruction. 

The situation is even more complicated for CLFA112, due to the presence of an additional As zigzag chain layer in a unit cell which lends four possible termination layers: a Ca/La layer above the As chain layer (Ca/La layer-1), an As zigzag chain layer, a Ca/La layer above the FeAs layer (Ca/La layer-2), and an FeAs layer.

We identified all four possible layers by characterizing topographic images (Figure 1b–e) as well as differential conductance maps (g(r, E) = dI/dV(r, E)) (SI.I). Figure 1b shows a topographic image of Ca/La layer-1, Figure 1c shows an As chain layer, Figure 1d shows a Ca/La layer-2, and, finally, Figure 1e shows an FeAs layer. (A detailed description of the identification of each layer can be found in the Supplementary Materials.) All layers showed 2 × 1 surface reconstructions in the topographic images, which was noticeable from the fact that the overall interval between the 1D-like atomic rows was twice the known As-As distance in all four discernible surfaces (Figure 1b–e). That is, we observed dimer rows rather than individual atomic rows in all the revealed surfaces. Notably, both Ca/La layers showed similar particle–hole asymmetric behavior in the spectra (insets of Figure 1b,d). The averaged spectrum for the As chain layer showed a broad V shape (inset of Figure 1c), while a clear gap feature was observed in the spectrum of the FeAs layer. The clearest superconducting gap with coherence peaks was observed only on Ca/La layer-2 (Figure 1d and Figure 3c). Fitting such spectra with a clear gap feature to a Dynes formula, the ratio was estimated as 2Δ/*k*_B_*T*_C_~5, suggesting that CLFA112 is a strong coupling superconductor.

**Figure 2:**
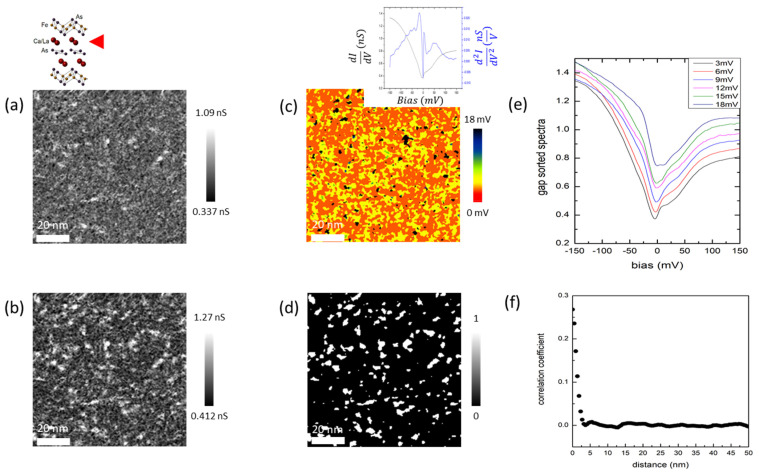
(**a**) A 92 nm × 92 nm image of g(r, E = 0 meV). (**b**) A 92 nm × 92 nm image of g(r, E = −12 meV). (**c**) A gap map produced using derivatives of the differential conductance in the same field of view (FOV) as in (**a**,**b**). (Inset) Averaged spectra of the differential conductances and their derivatives (An enlarged figure is shown in Figure S2b). (**d**) A mask image created to specify bright regions (impurities) in the image (**b**). (**e**) Plots of the gap-sorted dI/dV spectra, in nS (nanosiemens) units. (**f**) Azimuthally averaged cross-correlation coefficient between the black area mask of the gap map in (**c**) (shown in Figure S2c) and the impurity mask shown in (**d**).

Figure 2a,b are 92 nm × 92 nm images of the differential conductance maps for different energies: g(r, E = 0) and g(r, E = −12 meV) on the Ca/La layer-1. Many impurity-like, white, bright features are present in both images. The whole area shows a pseudogap-like suppression near the zero bias in the differential conductance spectra with a spatial variation noticeable from a map created by estimating a local gap size from g(r, E) in Figure 2c. Since there were no evident coherence peaks, we used derivatives of the differential conductance spectrum (d^2^I/dV^2^) of each point to estimate gap variations (Figure 2c). The inset shows an averaged differential conductance (black) and its derivative (blue). An inhomogeneous spatial-gap distribution is quite clear, and the lack of a direct correlation with topographic features suggests an influence from the sources residing on underlying layers. Figure 2e shows averaged gap-sorted spectra, revealing that the gap variation mainly comes from the spectral weight near E~10 meV. As the gap values increase, the averaged spectra show such trends, except for the spectrum with a gap value larger than 18 mV, where the gap is not as well-defined and hence the gap-detection algorithm [36] failed most likely (a black-colored region in Figure 2c). To identify the source of the inhomogeneity of the gap map [36], a mask depicted in Figure 2d where only areas with a relatively high conductance value in g(r, E = −12 meV) were marked as white. It is remarkable that studies on (Sr_1−x_La_x_)_3_Ir_2_O_7_ report a similar spectral feature on La dopants as well [37]. A correlation between the white area in Figure 2d and the black area in the gap map of Figure 2c is notable, and an azimuthally averaged cross-correlation (Figure 2f) between the gap-map mask in Figure 2d and the impurity mask in Figure S2c revealed a normalized cross-correlation coefficient [38] (defined in SI Equations (S1) and (S2)) of 0.268 (Figure S2d) at the center, with a rapidly decaying tail with a full width at half maximum of around 2.4 nm. The percentage of the white area in Figure 2d is 10.18% of the area of an entire field of view (FOV), which is comparable to a nominal concentration of La impurities (10%). Neither Figure 2c nor Figure 2d show a pronounced correlation with the topographic image. In general, dopants from the top surface appear as sharper features registered in a topograph. Combining this observation with the fact that positive La ions will tend to increase local electron density due to screening, we concluded that the bright feature in Figure 2d was due to La dopants on the underlying Ca/La layer-2, not on the Ca/La layer-1. Our correlation analysis indicates that the dopants disturb the superconductivity locally. This is similar to the case of Bi_2_Sr_2_CaCu_2_O_8+δ_, where oxygen dopants, which are essential to the superconductivity, appeared to suppress the gap locally [38].

**Figure 3:**
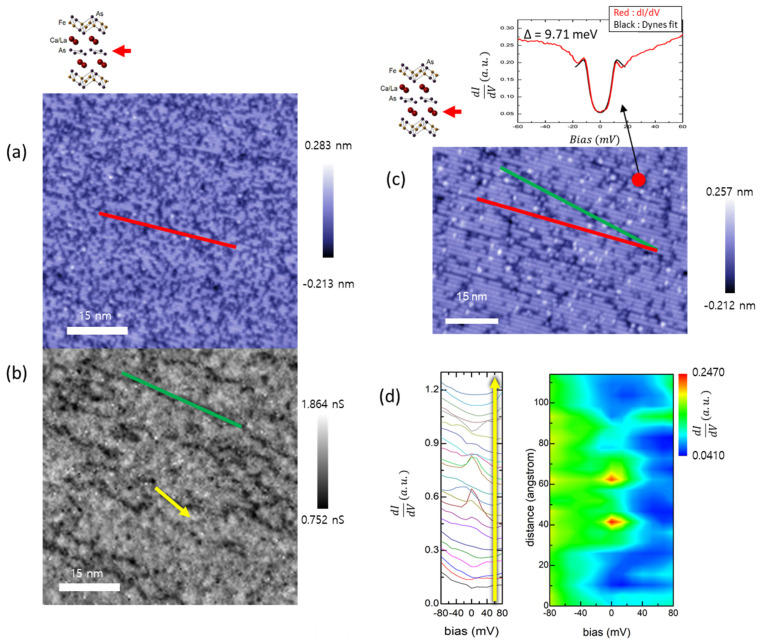
(**a**) Topographic image taken above the As chain layer (−200 mV/ 200 pA). (Red line: indication of surface-reconstruction line). (**b**) g(r, E = −100 mV) on the As chain layer. (Green line: indication of 1D features). (**c**) Green and red lines on the topographic image of the Ca/La-2 layer. The green line coincides with the crevice direction and the red line coincides with the surface-reconstruction direction. (Inset: point spectra on the red dot (red) and Dynes formula fit (black)). (**d**) Line profile along the yellow line in Figure 3b.

The spectral features of the As chain layer were quite distinct from those of the spectra of the other layers. Figure 3a is a topographic image of the As chain layer showing a 2 × 1 surface reconstruction. The averaged spectrum on the As chain layer (Figure 1c) did not show a narrow gap-like structure but displayed a broad suppression in low biases, unlike other spectra from the other three surfaces. Sparse one-dimensional lines, however, were visible in a conductance map over a range of biases. For example, g(r, E = −200 meV) in Figure 3b exhibits linear features (green line in Figure 3b) which are not from a simple surface reconstruction (red line in Figure 3a), since their angles differ from the 2 × 1 surface-reconstruction direction. Its origin was clear when we referred to a topographic image of the Ca/La layer-2 (Figure 1d and Figure 3c), whose orientation was registered with the surface-reconstruction direction in the As chain layer. The crevices (vacancies) found on Ca/La layer-2 precisely coincided with the one-dimensional features in Figure 3b, both in overall angle and shape. This observation was critical in determining the identity of the top surface as an As chain layer (SI.SI.4).

Since the As chain layer was predicted to be the source of possible topological superconductivity, we examined its local spectra by performing a high-resolution spectral mapping.

Remarkably, zero-bias conductance peaks (ZBCPs) were observed in the As chain layer. Figure 3d is a line profile of the differential conductance along the yellow line in Figure 3b, which corresponds to a crevice on Ca/La layer-2. ZBCPs mostly appear at the endpoints or right on top of the one-dimensional features and they were absent at other locations (Figure S4a). Considering the clear superconducting gaps observed on the crevices of the Ca/La layer-2 (Figure 3c), it is evident that our ZBCPs are due to an influence of the superconducting FeAs layer on the As chain layer through accidental one-dimensional crevices (vacancy lines) formed on the underlying Ca/La layer-2. Since our experimental setup was a vacuum tunneling with a tungsten tip, we can rule out the possibility of Josephson coupling as well as the Andreev bound state as an origin for the ZBCPs. In addition, impurity/vacancy-related bound states are unlikely, since the peaks observed were only at the exact zero bias. Considering that a usual proximity effect in a superconductor–metal system should exhibit a proximity gap, our observation of ZBCPs seems to suggest that the FeAs superconducting layer/As chain layer coupling is not a simple one. We cannot rule out the possibility that the topologically non-trivial edge states coupled to the superconducting pairs produce the ZBCPs, especially when natural 1D-wire-like patterns on the As chain layer coincide with occurrences of ZBCPs. The width of the ZBCPs were huge compared to other reported topological ZBCPs, which might be due to the modulation voltage (3 mV) used in our lock-in measurement.

**Figure 4:**
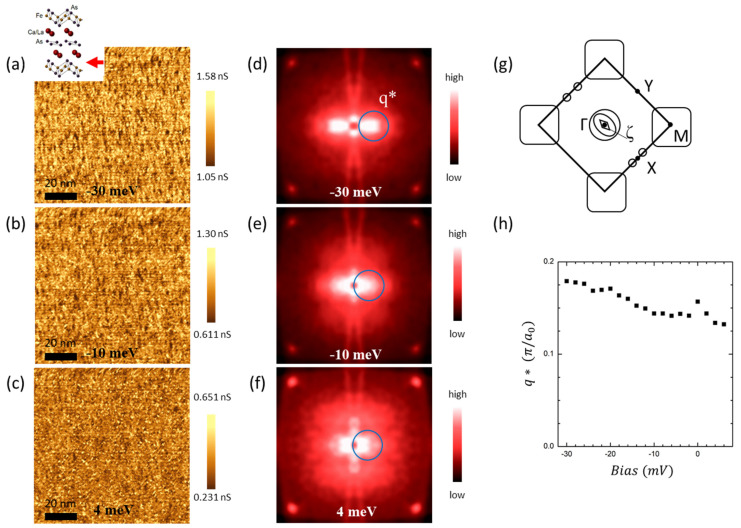
(**a**–**c**) A series of 88 nm × 88 nm images of g(r, E = −30 meV), g(r, E = −10 meV), and g(r, E = 4 meV). (**d**–**f**) Respective Fourier transforms of the conductance maps in (**a**–**c**). (**g**) Schematic Fermi surfaces. (**h**) A q*-versus-bias plot of the bright peaks designated by circles in the Fourier-transformed conductance maps shown in (**d**–**f**).

Figure 4a–f show g(r, E) and g(q, E). The g(q, E) images in Figure 4d–f are 2D Fourier transforms of g(r, E) images of the FeAs layer, with E = −30,−10 and 4 meV. Nematic features could be seen along a vertical direction. In g(q, E), the location of the peak (q*) in the blue circle disperses toward the center as the energy increases, while the non-dispersing sharp peaks at the corners are Bragg peaks. Figure 4g shows the schematic Fermi surface of CLFA112 [20]. The aforementioned peak in the blue circle is along the Γ–M direction, as shown in Figure 4g. Figure 4h is a plot of the peak’s location from g(q, E) versus energy. The dispersion shows a clear particle–hole asymmetry and a hole-like dispersion. Compared to ARPES measurements and theoretical calculations, the dispersion of the q* coincides with the ζ band of CLFA112 [20] near the Γ point (Figure 4g and Figure S5). The orbital character of the ζ band is known to be of *d_xz_* and *d_yz_* character, which is also consistent with the previous interpretation of nematic modulations in Fe-based 122 materials [32]. *d_xz_* and *d_yz_* orbitals are claimed to be the origin of nematicity in previous research on other samples [32]. However, in CLFA112, we observed that the nematicity coexists with SC. By adding one more Fe-based superconductor exhibiting a nematicity, our result suggests the universality of such broken symmetry in the Fe-based superconductor family.

## 4. Conclusions

In conclusion, we report a comprehensive atomic-scale study on CLFA112 for the first time. All four distinctive layers of CLFA112 were characterized. Depending on the terminating layers, we observed localized but well-defined SC gaps (Ca/La layer-2), inhomogeneous gaps without coherence peaks (Ca/La layer-1), broad semiconductor-like spectra with ZBCPs on 1D features (As chain layer), as well as a dispersing nematic modulation with spatially homogeneous gaps (FeAs layer). The localized ZBCPs on the As chain layers were clearly due to a proximity effect of the superconducting FeAs layer on the As chain layer, which is surprising, since a usual superconductor–metal system should exhibit a proximity gap, not a ZBCP under a vacuum tunneling. As of now, we cannot rule out the possibility that the ZBCPs were due to an induced SC gap on the topological edge states of the As chain layer, but the broad width of the ZBCPs suggests that further investigation (both theoretical and experimental) is necessary to verify this. One experimental challenge is the scarcity of sample-cleaving occurrences revealing an As chain structure as a terminating layer. The particle–hole asymmetric nematic dispersion is due to a hole-like pocket near Γ, which is consistent with the nematic modulations found in other Fe-based 122 materials [32], but we found that this nematic phase coexists with superconductivity in our case.

Fe-based 112 material seems to be very unique, not only in terms of providing information on how dopants influence superconductivity and in providing direct access to the FeAs layer, but also in producing highly unusual ZBCPs with 1D-wire-like distributions in the As chain layer. Still, it is not clear whether CLFA112 is a topologically non-trivial superconductor at the time of writing; however, the ZBCP on the As chain layer seems to be a candidate feature by means of which this question can be settled.

## Supplementary Materials

The following are available online at https://www.mdpi.com/article/10.3390/nano13040622/s1. Figure S1: (a) Topographic image (V = −100 mV and I = 100pA) taken above FeAs layer. (b) Averaged spectrum of conductance spectra (black) and derivative of conductance spectra(blue) taken on (a). (c) Gap map by differentiation of spectra. Figure S2: (a) Topographic image (V = −100 mV, I = 100 pA) taken above Ca/La layer-1. (b) Average of conductance spectra taken on (a). (c) 18 mV area masked with impurities in Figure 2c. (d) Cross-correlation map between (c) and Figure 2b. Figure S3: (a) Topographic image taken above Ca/La layer-2. The red area is where the gap-like spectra were observed. (b) Averaged differential conductance of the entire FOV(black) and the red area(red) of (a). (c) Point spectrum with much longer averaging time on crevices(red) and Dynes-formula-fit(black). Figure S4: (a) The mask (red) of the zero bias conductance peak regions overlapped with Figure 3b. (b) Averaged spectrum of the entire field of view (black) and the masked area(red) on (a). Figure S5: q* vs bias voltage plot and comparison with the ARPES measurement. Figure S6: Zoomed in inset figures from the main figure sets. The following references were cited in Supplementary Materials: [39,40,41].
