# Supplementary material for: Observations of Nematicity, Dopants, and Zero-Bias Conductance Peaks for the Ca0.9La0.1FeAs2 Superconductor
Source: Nanomaterials (Basel). 2023 Feb 4;13(4):622. doi: 10.3390/nano13040622 (PMC9962653; doi:10.3390/nano13040622)
Supplement: Supplementary file 1 [file nanomaterials-13-00622-s001.zip › nanomaterials-2157776-supplementary.pdf]

nanomaterials

Supplementary Materials

Observation of Nematicity, Dopants, and Zero-Bias Conductance Peaks for the $Ca_{0.9}La_{0.1}FeAs_2$ Superconductor

Jae-Joon Kim [1,2], Min Seok Park [1], Kyoung Seok Lee [1,2], Sang Hyun Joo [1,2], Jung Hoon Yoo [1], Dilip Bhoi [3], Byeong Hun Min [3], Kee Hoon Kim [3] and Jinho Lee [1,*]

1 Department of Physics and Astronomy, Seoul National University, Seoul 08826, Republic of Korea
2 Samsung Electronics Semiconductor R&D Center, Hwaseong-si 18448, Republic of Korea
3 Center for Novel States of Complex Materials Research, Department of Physics and Astronomy, Seoul National University, Seoul 08826, Republic of Korea
* Correspondence: jinholee@snu.ac.kr

1. I. Identification of surfaces of CLFA112

Due to the number of possible terminating layers and the scarcity of the occurrence of revealing certain layers by cleaving, we relied on the spectroscopic/topographic signatures measured on numerous cleaved surfaces over years and comparison of them to those from the other previous studies on 122 Fe based superconductors and dopants studies on other materials (e.g. cuprates, La doped iridates etc.) to identify 4 distinguished terminating layers of CLFA112. All spectroscopic maps and topographic images presented in the main text and the SI were taken at 4.2 K and 1 GOhm junction resistance unless specified otherwise. We confirmed that there was no systematic bias offset by examining I-V spectra (crossing point of I-V curves being zero bias) obtained as a byproduct of the spectroscopic mapping.

1. FeAs layer

FeAs layer is the primary layer of the superconductivity in Fe Based superconductors(FeSC's). As FeSC is widely accepted as an s-wave superconductor[1,2], we can expect the superconducting gap will show more or less a homogeneous distribution if we measure the superconducting gap directly on FeAs layer. Ming *et al.* [3] showed that 2x1 surface reconstruction of top As atoms of FeAs layer. A topographic image on FeAs layer in SI figure 1a displays a similar 2x1 surface reconstruction. The black curve in SI figure 1b is a conductance spectrum averaged over the entire FOV in SI figure 1a. The entire area shows gap-like differential conductance spectra from which we were able to produce a gap-map from the g(r, E). Since there are no evident coherence peaks, we numerically obtained a derivative on differential conductance (dI/dV) spectrum on each point where peaks related to slope changes in dI/dV (SI figure 1b) and we estimated the relative gap sizes from the bias values where those peaks occur. SI figure 1c is a resulting gap-map. An average spectrum of the differential conductance (black) as well as an average derivative of the differential conductance (blue) is shown in SI Figure 1(b). Peaks from d^2I/dV^2 can be seen clearly in the blue spectrum. Since the gap values in SI figure 1c are not from a conductance spectrum, we cannot use those as an absolute gap value but they rather represent relative values and their distribution. In SI figure 1c, gap values are quite homogeneous. An offset in the spectrum is due to the charge polarity of the top surface. FeAs layer is charged negatively and to make the net bias voltage zero, a slight positive bias is needed which causes the bias offset in spectra. From the topographic image, the differential conductance spectrum, and the gap distribution, we concluded this layer as was a FeAs layer.

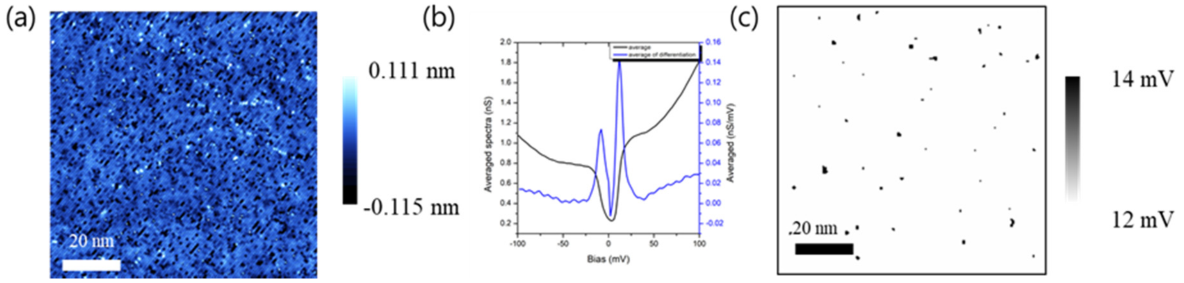

Figure S1. (a) Topographic image (V = -100 mV and I = 100pA) taken above FeAs layer. (b) Averaged spectrum of conductance spectra (black) and derivative of conductance spectra(blue) taken on (a). (c) Gap map by differentiation of spectra.

2. Ca/La layer-1(above As chain layer)

In the case of Ca-122 material, the Ca layer is predicted to show 2x1 surface reconstruction[4]. If we measure a differential conductance above the Ca/La layer, the spectra might exhibit an inhomogeneous distribution and the particle-hole symmetry unlike those on FeAs layer. The topographic image (SI figure 2a) shows a 2x1 surface reconstruction. Black lines in SI figure 2b is an averaged dI/dV spectrum of the entire FOV of the SI figure 2a which shows a clear particle-hole asymmetric behavior. Still, there is a gap-like feature around E < 20 meV, and it was possible to make a gap-map from those point spectra. As in the case of the FeAs layer, the coherence peaks are not evident and we resorted to the same method we used in the FeAs layer's case. Figure 2c is gap-map from the derivatives of the conductance spectra and figure 2e is averaged gap-sorted spectra. Unlike the FeAs layer's case, the gap-map shows a clear inhomogeneous distribution of its values providing an evidence that this layer is not the FeAs layer. Gap values ranged from 3 meV to 18 meV. In the gap-sorted spectrum, the gap-like features can be seen up to an estimated gap value of 15 meV. In the gap sorted spectrum with the gap value larger than 15 meV, the gap-like feature becomes weak and the spectrum is different from the others. That is due to the gap-value-detection algorithm and we can regard such an area as a gapless area where superconductivity is suppressed. Figure 2b is g(r, E = -12 meV) and there are features appearing as white speckles. Figure 2d is a mask of the white features in figure 2b. The white area in figure 2b shows a correlation with that of the SI figure 2c where the area with the gap value 18 meV in the gap map is marked as white color in the mask. The cross-correlation map between figure 2d and SI figure 2c is SI figure 2d and an azimuthally averaged spectral weight of the SI figure 2d is shown in figure 2f. We calculated the normalized cross correlation coefficient according to the following formulae:

$$C_{f,g}(\vec{R}) = \frac{\int [f(\vec{r}) - \bar{f}] \times [g(\vec{r} + \vec{R}) - \bar{g}] d^2 r}{\sqrt{A_{f,f}(0) A_{g,g}(0)}} \quad (1)$$

$$A_{f,f}(\vec{R}) = \int [f(\vec{r}) - \bar{f}] \times [f(\vec{r} + \vec{R})] - \bar{f}] d^2 r \quad (2)$$

where $C_{f,g}(\vec{R})$ is the normalized cross correlation between the functions *f* and *g* as a function of $\vec{R}$ and $A_{f,f}(\vec{R})$ is the auto-correlation of a function *f*.

The correlation coefficient is 0.268 at the center of SI figure 2d and it decays to zero rapidly. Almost zero correlation after 2nm in distance. The percentage of the white area in figure 2d is 10.14 % which is comparable to the nominal doping level of La (10%) atoms and it is evident that those white features are not originated from the topographic image. We concluded that the white area in figure 2d is due to La dopants on an underneath Ca/La layer. It seems contradictory that the dopants can hamper a superconductivity locally but a similar phenomenon is already reported in the case of $Bi_2Sr_2CaCu_2O_{8+x}$. Oxygen doping is essential to the superconductivity in $Bi_2Sr_2CaCu_2O_{8+x}$ and yet the superconductivity is suppressed locally by oxygen dopants[4]. A topographic image, inhomogeneous gap distribution and the fact that dopants are not originated from the top surface leading to a conclusion that this surface is the Ca/La layer above As chain layer.

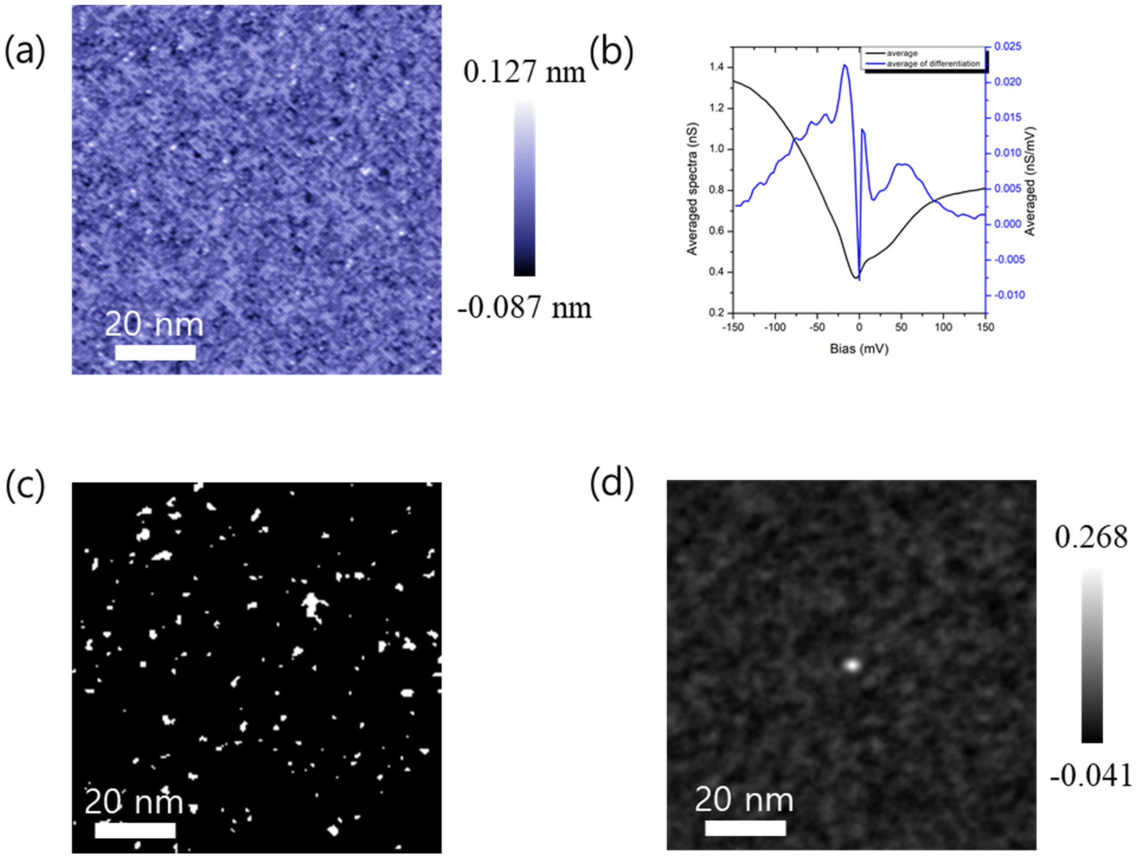

Figure S2. (**a**) Topographic image (V = -100 mV I = 100pA) taken above Ca/La layer-1. (**b**) Average of conductance spectra taken on (**a**). (**c**) 18 mV area masked with impurities in Figure 2c. (**d**) Cross-correlation map between (**c**) and Figure 2b.

3. Ca/La layer-2(above FeAs layer)

Similar to the Ca/La layer above As chain layer, Ca/La layer above the FeAs layer showed a 2x1 surface reconstruction. SI figure 3a is a topographic image and the black curve in SI figure 3b is an averaged spectrum. The averaged spectrum is different from the aforementioned surfaces. The uniqueness of this surface is that there are gap-like features on crevices of the topographic image. Points exhibiting Gap-like features are depicted as red points in SI figure S3a and those points' average is plotted as the red curve in SI figure 3b. SI figure S3c is a point spectrum on crevices with a much longer averaging time and it shows a clear superconducting gap feature. Such spectra exhibit a more pronounced p-h symmetric shape than the spectrum taken above the FeAs layer which is probably due to the lack of surface reconstruction of the fully revealed FeAs layer. Namely a direct measurement of the SC gaps on the bare FeAs layer is hampered by the surface reconstruction of the FeAs layer. In the case of Ca/La layer-2, however, there might be no surface reconstruction in the underlying FeAs layer. Remarkably, when we probe the crevices of Ca/La layer-2, we could avoid effect from the top Ca/La layer as well which resulted in a clear SC gap dI/dV. Distinct averaged spectra between the spectrum on crevices and the other area indicate that the surface belongs to the Ca/La layer just above the FeAs layer.

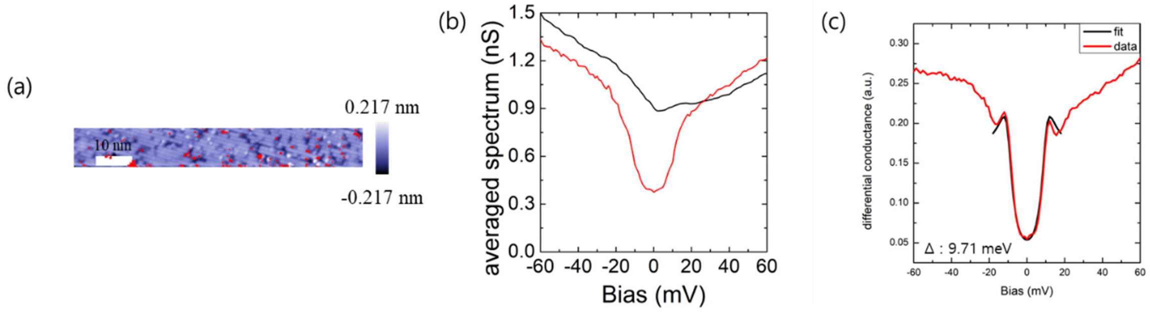

Figure S3. (**a**) Topographic image taken above Ca/La layer-2. The red area is where the gap like spectra were observed. (**b**) Averaged differential conductance of the entire FOV(black) and the red area(red) of (**a**). (**c**) Point spectrum with much longer averaging time on crevices(red) and Dynes-formula-fit (black).

4. As chain layer

The remaining possible termination surface is the As chain layer. A spectrum on the As chain layer was remarkably distinct from others. Figure 3a is a topographic image which shows a 2x1 surface reconstruction overall while numerous vacancy-like black regions are evident. The occurrence of such surface was extremely rare (less than 10%) and the topographic feature suggests highly unstable nature (the areal portion of the vacancies is comparable to the areal portion of the remaining top layer atoms). The average spectrum (Inset of figure 1c) shows a semiconductor-like broad suppression near the zero bias which is highly distinctive from the other 3 surfaces. In figure 3b, g(r, E = -200 meV) shows there are one-dimensional(1D) features (green line) and compared to the surface reconstruction of topographic image (red line in figure 3a), those two 1D features stretch in a different angle. Compared to the topographic image of Ca/As layer-2 (figure 3c), crevices on Ca/As layer-2 coincide with 1-dimensional features in figure 3b. Such 1D features(green line) which are only detectable in the spectroscopic map, not in the topography clearly suggest the surface is on top of the Ca/La layer-2. From a comparison of g(r, E) and topographic image, this layer is not from the Ca/La layer-1 either (SI I-2). The only remaining possibility is the As chain layer.

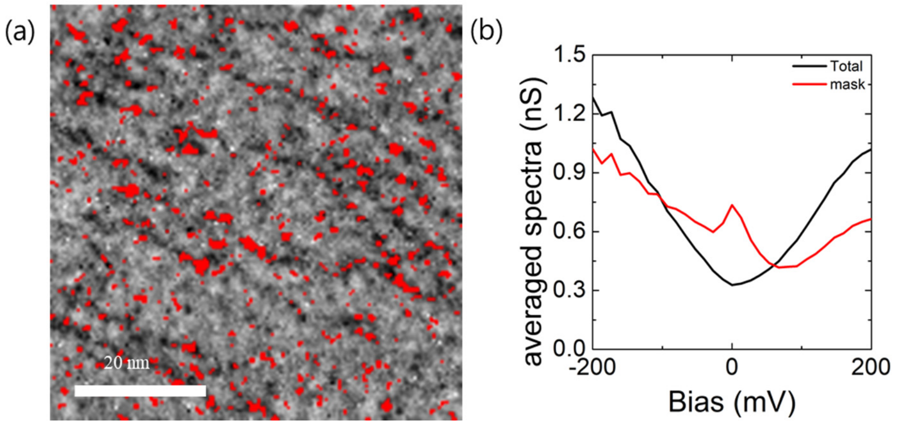

Figure S4. (**a**) The mask (red) of the zero bias conductance peak regions overlapped with Figure 3b. (**b**)Averaged spectrum of the entire field of view (black) and the masked area(red) on (**a**).

2. Dispersion of the q*

We compared the dispersion of the nematic feature shown in Figure 4h to the previously reported bands near the Γ point and the result is shown in figure S5b. We needed to displace our result in energy to find the best match between the dispersions slopes of the STM and the ARPES results. This displacement in energy is probably due to the difference between the nominal and the actual doping levels.

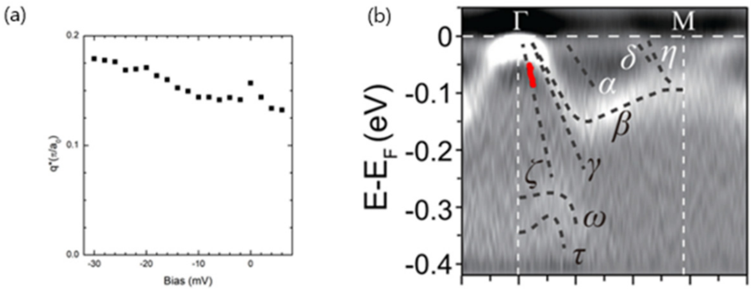

Figure S5. q* vs bias voltage plot and comparison with the ARPES measurement.

(**a**) Plot of q* in Figure 4h (using amplitude of gradient) versus bias voltage. It shows a clear particle hole asymmetric and hole-like dispersion. (**b**) Comparison between our STM result (red dots) and ARPES measurement (background). ARPES measurement was taken with circularly polarized photon with energy 104 eV at 15-20 K. Dispersion of q* from our result matches well with ζ band in ARPES measurement when slightly displaced in bias. ARPES result reproduced from Li et al [5].

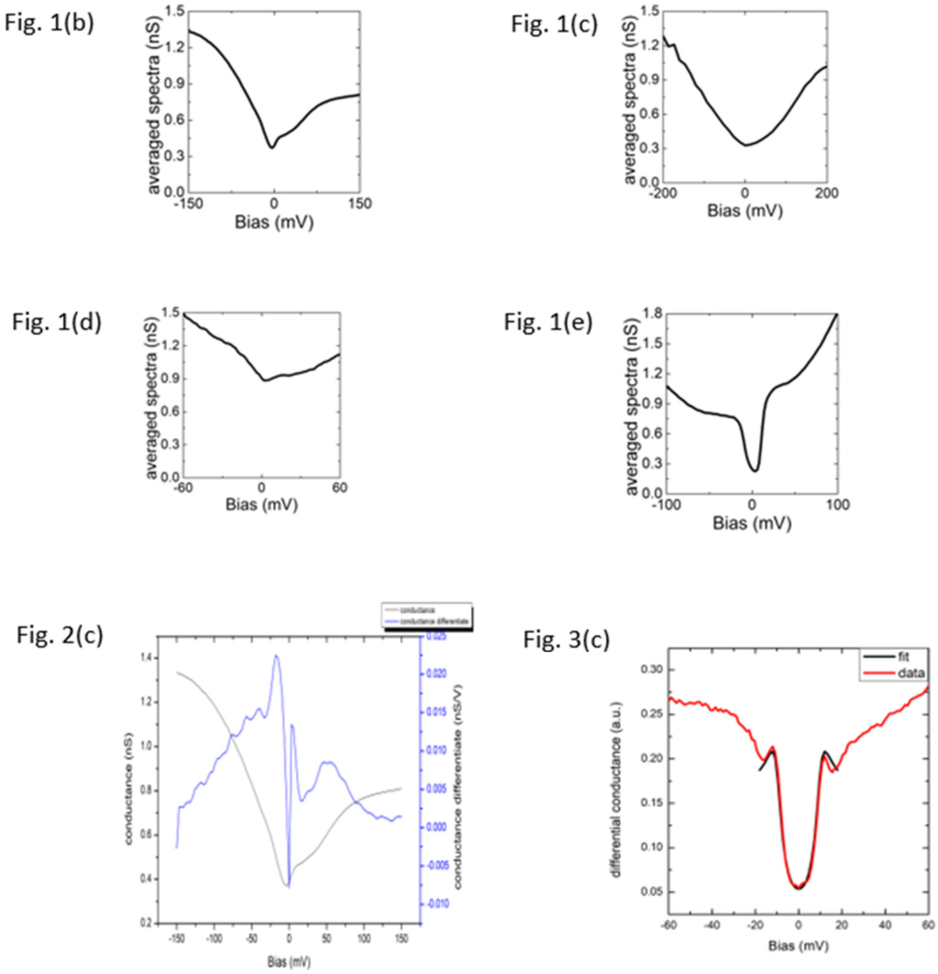

Figure S6. Zoomed in inset figures from the main figure sets.

Reference

(1) Johnston, D. C. The Puzzle of High Temperature Superconductivity in Layered Iron Pnictides and Chalcogenides. *Adv. Phys.* **2010**, *59* (6), 803–1061. https://doi.org/10.1080/00018732.2010.513480.

(2) Hirschfeld, P. J.; Korshunov, M. M.; Mazin, I. I. Gap Symmetry and Structure of Fe-Based Superconductors. *Reports Prog. Phys.* **2011**, *74* (12). https://doi.org/10.1088/0034-4885/74/12/124508.

(3) Chuang, T.-M.; Allan, M. P.; Lee, J.; Xie, Y.; Ni, N.; Bud'ko, S. L.; Boebinger, G. S.; Canfield, P. C.; Davis, J. C. Nematic Electronic Structure in the "Parent" State of the Iron-Based Superconductor Ca(Fe(1-x)Co(x))2As2. *Science* **2010**, *327* (5962), 181–184. https://doi.org/10.1126/science.1181083.

(4) Gao, M.; Ma, F.; Lu, Z.-Y.; Xiang, T. Surface Structures of Ternary Iron Arsenides AFe2As2 (A=Ba, Sr, or Ca). *Phys. Rev. B* **2010**, *81* (19), 193409. https://doi.org/10.1103/PhysRevB.81.193409.

(5) Li, M. Y.; Liu, Z. T.; Zhou, W.; Yang, H. F.; Shen, D. W.; Li, W.; Jiang, J.; Niu, X. H.; Xie, B. P.; Sun, Y. Significant Contribution of As 4 p Orbitals to the Low-Lying Electronic Structure of the 112-Type Iron-Based Superconductor Ca 0.9 La 0.1 FeAs 2. Phys. Rev. B 2015, 91 (4), 045112.
